# Anion exchange HPLC monitoring of mRNA *in vitro* transcription reactions to support mRNA manufacturing process development

**DOI:** 10.3389/fmolb.2024.1250833

**Published:** 2024-03-07

**Authors:** Emma N. Welbourne, Kate A. Loveday, Adithya Nair, Ehsan Nourafkan, Jixin Qu, Ken Cook, Zoltán Kis, Mark J. Dickman

**Affiliations:** ^1^ Department of Chemical and Biological Engineering, University of Sheffield, Sheffield, United Kingdom; ^2^ ThermoFisher Scientific, Hemel Hempstead, United Kingdom; ^3^ Department of Chemical Engineering, Imperial College London, London, United Kingdom

**Keywords:** mRNA, anion exchange HPLC, *In vitro* transcription, mRNA vaccines/therapeutics, process development

## Abstract

mRNA technology has recently demonstrated the ability to significantly change the timeline for developing and delivering a new vaccine from years to months. The potential of mRNA technology for rapid vaccine development has recently been highlighted by the successful development and approval of two mRNA vaccines for COVID-19. Importantly, this RNA-based approach holds promise for treatments beyond vaccines and infectious diseases, e.g., treatments for cancer, metabolic disorders, cardiovascular conditions, and autoimmune diseases. There is currently significant demand for the development of improved manufacturing processes for the production of mRNA therapeutics in an effort to increase their yield and quality. The development of suitable analytical methods for the analysis of mRNA therapeutics is critical to underpin manufacturing development and the characterisation of the drug product and drug substance. In this study we have developed a high-throughput, high-performance liquid chromatography (HPLC) workflow for the rapid analysis of mRNA generated using *in vitro* transcription (IVT). We have optimised anion exchange (AEX) HPLC for the analysis of mRNA directly from IVT. Chromatography was performed in under 6 min enabling separation of all of the key components in the IVT, including nucleoside triphosphates (NTPs), Cap analogue, plasmid DNA and mRNA product. Moreover, baseline separation of the NTPs was achieved, which facilitates accurate quantification of each NTP such that their consumption may be determined during IVT reactions. Furthermore, the HPLC method was used to rapidly assess the purification of the mRNA product, including removal of NTPs/Cap analogue and other contaminants after downstream purification, including solid phase extraction (SPE), oligo deoxythymidine (oligo-dT) affinity chromatography and tangential flow filtration (TFF). Using the developed method excellent precision was obtained with calibration curves for an external mRNA standard and NTPs giving correlation coefficients of 0.98 and 1.0 respectively. Intra- and inter-day studies on retention time stability of NTPs, showed a relative standard deviation ≤ 0.3% and ≤1.5% respectively. The mRNA retention time variability was ≤0.13%. This method was then utilised to monitor the progress of an IVT reaction for the production of Covid spike protein (C-Spike) mRNA to measure the increasing yield of mRNA alongside the consumption of NTPs during the reaction.

## 1 Introduction

mRNA has recently emerged as a new class of therapeutic, as demonstrated by the development and approval of two highly efficacious vaccines based on mRNA sequences encoding for a modified version of the SARS-CoV-2 spike protein (Corbett et al., 2020; Polack et al., 2020). Furthermore, RNA-based approaches have potential for treatments beyond vaccines and infectious diseases as treatments for cancer, metabolic disorders, cardiovascular conditions, and autoimmune diseases. During the enzymatic manufacturing process of mRNA vaccines/therapeutics, incomplete mRNA products are generated in conjunction with other potential impurities such as double stranded (ds)RNA. Furthermore, during manufacturing and storage, RNA and RNA therapeutics can be degraded by exposure to heat, hydrolysis, oxidation, light and ribonucleases. The development of analytical methods for the analysis of mRNA therapeutics is critical to underpin manufacturing development. Analytical methods are also required to assess batch to batch manufacture and process repeatability, as well as the quality of mRNA produced. Furthermore, validated analytical methods are required to support the relevant phase of clinical development, regulatory submission requirements or to support ongoing quality control of the approved product. Current analytical methods available to characterise RNA therapeutics are limited and the development of methods for the analysis of large RNA (>1,000 nucleotides), including mRNA vaccines, is challenging. Therefore, there is currently significant demand for the development of new and improved analytical methods for the characterization of mRNA therapeutics.

The mRNA manufacturing process centres on the synthesis of the mRNA drug substance. Typically, mRNA can be synthesized using both chemical and enzymatic methods. Baronti el al. have reviewed the most suitable methods for RNA preparation, presenting their advantages, disadvantages and their latest advances (Baronti et al., 2018). Chemical synthesis of RNA is routinely performed using solid phase synthesis, with an upper size chain limit of approximately 100 nucleotides after repetitive yields of >99%, while physiological mRNAs are typically 1,000–10,000 nucleotides. Although mRNA’s length is restrictive for its chemical synthesis, enzymatic *in vitro* transcription (IVT) is considered a simple and inexpensive procedure for mRNA synthesis, which can yield products of variable sizes in Gram quantities (Beckert et al., 2011; Sahin et al., 2014). In a commercial manufacturing process, sufficient amounts of functional pharmaceutical mRNA are typically synthesized in a cell-free system by IVT.

Plasmid DNA (pDNA), isolated and purified from bacterial cells and subsequently linearised by an appropriate restriction enzyme is commonly used as the DNA template for IVT reactions. Typical plasmid vectors contain a T7 promoter sequence upstream of multiple cloning sites. Furthermore, a poly(A) tail sequence, 5′ and 3′ untranslated regions are included in the DNA template design (Hadas et al., 2019; Tusup et al., 2019). In addition, a DNA template can be generated using a PCR product consisting of a T7 promoter at the 5′ end (Hadas et al., 2019). Typically, the poly(A) tail is incorporated in the initial DNA plasmid to be transcribed (Grier et al., 2016; To and Cho, 2021). Alternatively, mRNAs can be generated without a 3’ poly(A) tail using “tailless” pDNA template and a further poly(A) tailing step is performed post-transcriptionally. Under these conditions a poly(A) polymerase can be used to add poly(A) tails of approximately 80–160 nucleotides.

IVT reactions are commonly performed using highly processive, single-subunit, bacteriophage DNA dependent RNA polymerases, and have been widely established as a cost-effective and scalable mRNA manufacturing process (McNamara et al., 2015). Bacteriophage T3, T7, and SP6 DNA dependent RNA polymerases are single polypeptide chains that require only Mg^2+^ as a cofactor and run off the DNA template after several transcription reactions (Sahin et al., 2014; Baronti et al., 2018). In the IVT reaction the linearized pDNA template, containing an RNA polymerase promoter sequence, is typically incubated with the DNA dependent RNA polymerase, nucleoside triphosphates (NTPs), RNase inhibitor and inorganic pyrophosphatase. This method has been widely employed for large scale IVT mRNA manufacturing. Furthermore, a variety of mutant DNA dependent RNA polymerases have been used in an approach to incorporate modified nucleotides (Meyer et al., 2015; Duffy et al., 2020) and reduce the synthesis of dsRNA impurities (Dousis et al., 2023). Optimisation of the IVT reactions and mRNA manufacturing process enables significant increases in mRNA yield and quality of the mRNA drug substance (e.g., reduced impurities and increases integrity of the mRNA). High quantities of mRNA can be produced in a few hours.

The application of high-performance liquid chromatography (HPLC) has previously been focussed on the purification of RNA generated using IVT reactions, including short RNA transcripts (<40 nt) synthesised for nuclear magnetic resonance structural studies (Anderson et al., 1996). The addition of the hammerhead ribozyme into the sequence has allowed workers to overcome the length limitation of short RNAs in HPLC purification (Shields et al., 1999). RNA transcription reaction mixtures have been directly purified by weak anion exchange (AEX) fast protein liquid chromatography (FPLC) to remove free nucleotides, short abortive transcripts, linearized plasmids, and enzymes from the desirable transcripts within 4 h (Easton et al., 2010). Strong AEX (Mono Q) FPLC for transcript purification and in the studies of transcription reactions has also been demonstrated (Koubek et al., 2013). Analysis of oligonucleotides (OGNs) and RNA under denaturing conditions in conjunction with AEX HPLC has been performed in a variety of different systems in an approach to remove secondary/tertiary structures in OGNs and RNA (Thayer et al., 2005; Cook and Thayer, 2011; Kanavarioti, 2019; Sun et al., 2020).

Optimisation of the mRNA manufacturing process enables significant increases in mRNA yield and quality of the mRNA drug substance (e.g., reduced impurities and increased integrity of the mRNA). Rapid analysis of the mRNA generated in the manufacturing process, using on-line or near at-line analytical methods, is key to underpin process optimisation. In this study we have developed a rapid method for the analysis of IVT reactions using AEX HPLC. This method enables quantification of NTPs/Cap analogue, mRNA and residual DNA with separations performed in <6 min. This method has been successfully used to measure mRNA yield, NTPs (consumption) and mRNA purification for high throughput studies providing important information to support mRNA manufacturing process development and downstream processing.

## 2 Materials and methods

### 2.1 *In vitro* transcription

mRNA transcripts were prepared using *in vitro* transcription. eGFP and C-Spike plasmid DNA was provided by Genscript, NLuc plasmid DNA was provided by Aldevron. *In vitro* transcription reactions utilised template DNA (linearised plasmid), at 2. E−05 mM. DNA-dependent RNA polymerase of T7 bacteriophage (Roche) and ATP, CTP, GTP and UTP (Roche) in an equimolar ratio at 10 mM concentration were added to the reaction mixture. The reaction was further supplemented with the standard reaction buffer provided by the enzyme manufacturer. Inorganic pyrophosphatase (Roche) at 2.9E-03 mM was added to the reaction mixture to prevent magnesium pyrophosphate precipitation. RNase inhibitor (Roche) was added at 2.1E-04 mM to maintain RNase free environment in the reaction mixture. The reaction was incubated at 37°C for 2 hours. After incubation, the reaction was quenched by adding 200 mM EDTA. Following IVT, RNA was purified by solid phase extraction using silica columns as previously described (Nwokeoji et al., 2017). RNA concentrations were determined using a NanoDrop™ 2000c spectrophotometer (ThermoFisher Scientific) by absorbance at 260 nm normalized to a 1.0 cm (10.0 mm) path.

### 2.2 Tangential flow filtration (TFF)

A benchtop cross-flow TFF system (KrosFlo^®^ KR2i, Repligen) and hollow fiber (HF) filter modules (300 kDa mPES, 20 cm^2^) were used to separate the mRNA from unreacted NTPs in the IVT reaction mixture. All experiments were carried out at a feed flowrate of 10 mL/min and room temperature of 20.0°C ± 0.5°C. Crude IVT samples were diluted 16 times in 40 mM HEPES buffer (pH 7), before feeding through the TFF. The mRNA separation was accomplished by 4X concentration followed by 5X dia-filtration volumes of fresh buffer. At the end of process, the HF filter was washed with 20 mL of fresh buffer followed by 20 mL RNase-free water to recover any remaining mRNA from the TFF system.

### 2.3 mRNA oligo-dT chromatography purification

mRNA of interest in crude IVT was isolated and purified by oligo-dT chromatography using a 1 mL monolithic column (CIMmultus Oligo dT18; Sartorius, Gottingen, Germany) on an AKTA PCC (Cytiva, Uppsala, Sweden). All steps were performed at room temperature and UV sensors at 280 nm were used for all stages. The column was equilibrated in 20 mL loading buffer (250 mM NaCl, 50 mM sodium phosphate, 5 mM EDTA, pH 7.0) at a flow rate of 2 mL/min and the samples were loaded at 1 mL/min in 10 mL loading buffer. This was followed by a wash step in 20 mL loading buffer at 2 mL/min. The column bound fraction was eluted using 10 mL nuclease-free water at 2 mL/min. Chromatograms were generated with the UNICORN software (Cytiva, Uppsala, Sweden).

### 2.4 Anion exchange high-performance liquid chromatography (AEX HPLC)

IVT reaction samples (20 μL) were quenched by addition of 2 μL of 200 mM EDTA. Quenched samples were diluted 100–500 fold with RNase-free water to reach the final mRNA concentration 0.2–100 ng/μL. Injection volume was 5 µL. Samples were analysed by AEX on a U3000 HPLC system using a DNAPac PA200 column (50 mm × 2.1 mm I.D. Thermo Fisher Scientific). Chromatograms were generated using UV detection at a wavelength of 260 nm. The chromatographic analysis was performed using the following conditions: 10 mM NaOH in mobile phase A and 10 mM NaOH, 2 M NaCl in mobile phase B. Samples were analysed using the following gradient: 0%–15% mobile phase B over 2 min, 15%–55% mobile phase B over 1 min, 55%–65% mobile phase B over 1 min and 65%–100% over 1 min. This was performed at a flow rate of 0.5 mL/min and a temperature of 25°C. Standard curves for individual NTPs were prepared by a serial dilution of each reagent from 100 mM stock in RNase-free water to a final concentration range of 0.5–100 μM (NTPs). A standard curve for mRNA was prepared by a serial dilution from 1 mg/mL stock to a final concentration range of 0.2–100 ng/μL. The slope and intercept of the standard curve were calculated by fitting with a linear regression function. Limits of detection (LOD) and limits of quantification (LOQ) were determined using the Chromeleon ICH LOD LOQ (SN) processing method in Chromeleon 7.2. The following variables were chosen in the method Component Table: Cal. Type = Lin. WithOffset, Measure_Noise = Current, LOQ_Ratio = 10, LOD_Ratio = 3, Noise_Start = 5.5 min and Noise_End = 7.5 min.

## 3 Results and discussion

### 3.1 Optimisation of AEX HPLC

Initial work focussed on the optimisation of the mobile phases utilised in conjunction with AEX HPLC to enable the rapid separation of NTPs, mRNA and plasmid DNA in a single analysis. AEX chromatography was performed on DNAPac PA200 (2.1 mm × 50 mm) to facilitate the use of short gradients and enable high throughput analysis of mRNA from IVT reactions. A number of different mRNA sequences were synthesised using IVT, including eGFP (∼930 nt), COVID spike protein (C-Spike ∼4,300 nt) and NanoLuc (NLuc, ∼870 nt). Following IVT and purification of the mRNA, further characterisation was performed using capillary electrophoresis for sizing of the mRNA and analysis of mRNA integrity (see Supplementary Figure S1). The results confirm the expected size of the mRNAs and purification of intact mRNA. Furthermore direct RNA sequence mapping using LC MS in conjunction with partial RNase T1 digests was performed to confirm the sequence of the mRNAs [Vanhinsbergh et al., 2021]. The results show >90% sequence coverage was obtained based on unique oligoribonucleotides. These results confirm the sequence, size and integrity of the mRNAs used in this study.

**FIGURE 1:**
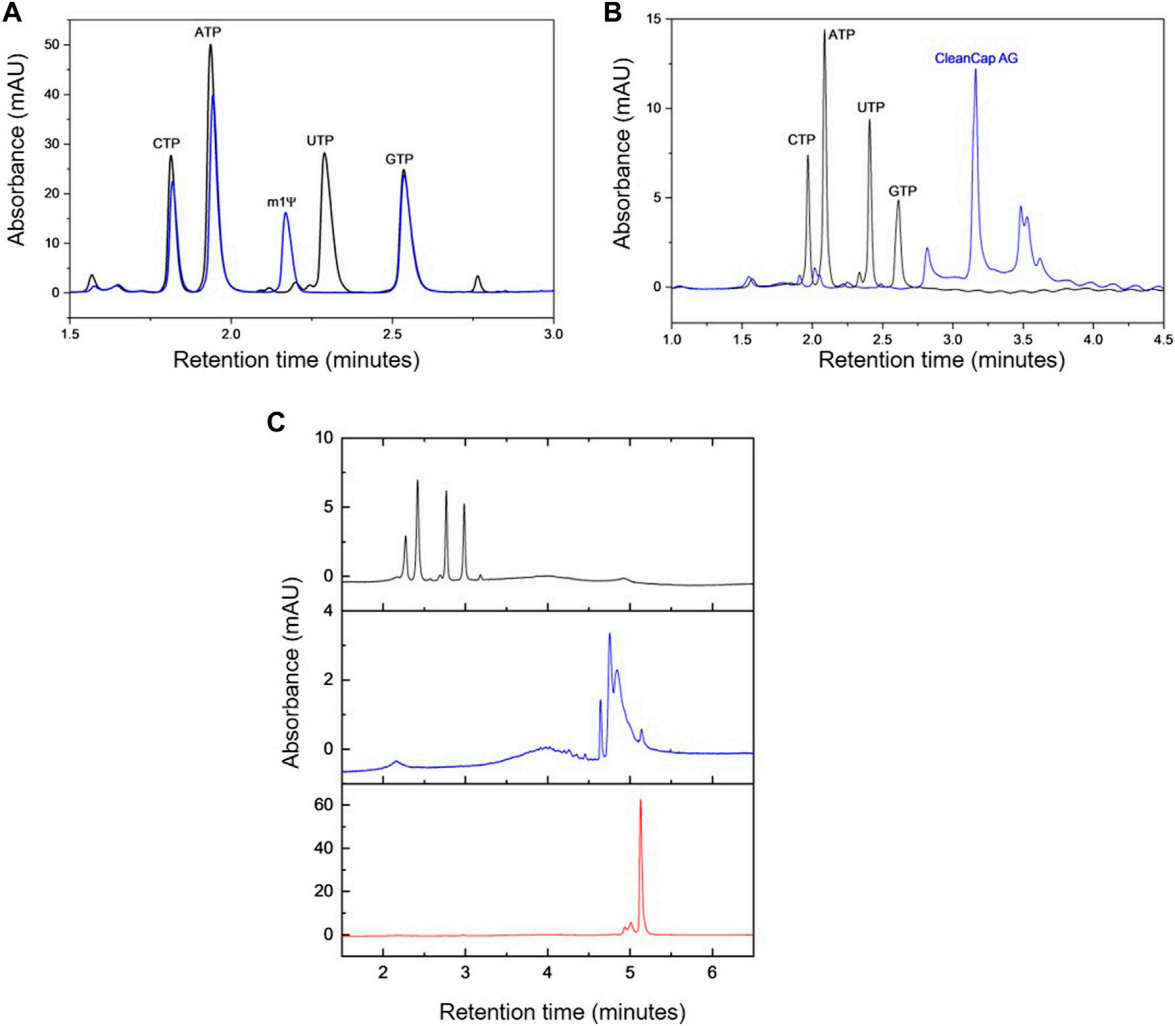
AEX HPLC analysis. **(A)** AEX chromatograms generated from a mixture of NTPs (black) and a mixture of NTPs where UTP is replaced by N1-methylpseudo-UTP (m1Ψ, blue). **(B)** AEX chromatograms produced by a mixture of NTPs (black) and CleanCap^®^ AG (m7G (5′)ppp (5′) (2′OMeA)pG) (blue). **(C)** Comparative analysis of AEX chromatograms from a mixture of NTPs (black), eGFP plasmid DNA (blue) and eGFP mRNA (red).

Initial work was performed using mobile phases at pH 8.0 (buffering with 25 mM Tris-HCl), in conjunction with increasing sodium chloride concentration for elution. This resulted in the incomplete separation of all of the NTPs (see Supplementary Figure S2A). Moreover, under these conditions incomplete and variable elution of the mRNA was observed from the stationary phase. Therefore, in an approach to further optimise the AEX HPLC, mobile phases were prepared at pH ≈ 11.95 using 10 mM sodium hydroxide. The resulting chromatogram demonstrating the baseline separation of the NTPs, including N1-methylpseudo-UTP (m1Ψ-UTP), is shown in Figure 1A. By performing the separation at pH 11.95, the ionisation state of the nucleobases of guanosine and uridine is changed (deprotonated at N1 and N3 respectively) as pH is increased from 8.0 to 11.95. Furthermore, by moving to pH 11.95, this resulted in complete and consistent elution of mRNA and no mRNA was retained on the column. Previously, AEX at high pH has been used to analyse RNA (sgRNA, tRNA and mRNA) and demonstrated the improved resolution of RNA under these conditions by removing the secondary/tertiary structures present and maintaining stability of the RNA under these chromatographic conditions (Kanavarioti, 2019). Following mobile phase optimisation, further work was performed to optimise the AEX gradient and enable rapid separation of the NTPs, cap analogue, mRNA and pDNA in a single analysis (see Figures 1B, C). The results show that separation of the NTPs, cap analogue, mRNA and pDNA can be achieved <6 min using the optimised gradient.

**FIGURE 2:**
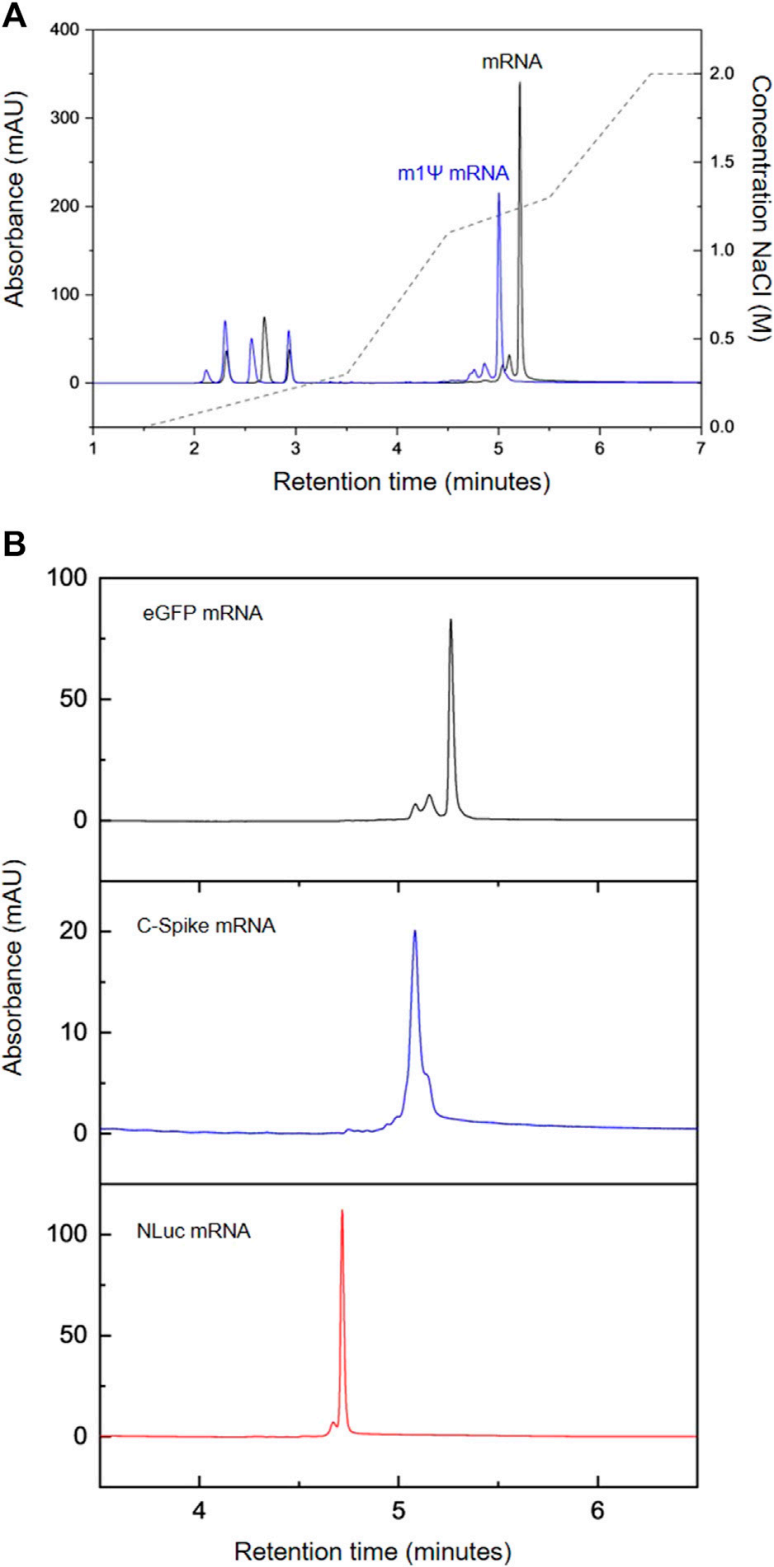
AEX HPLC analysis of IVT mRNA. **(A)** AEX analysis of IVT reactions producing modified eGFP mRNA (m1Ψ, blue) and unmodified eGFP mRNA (black) **(B)** AEX analysis of a range of different mRNA. eGFP (black), C-Spike (blue) NLuc (red).

mRNA vaccines/therapeutics are typically manufactured using chemically modified UTP such as m1Ψ-UTP. Therefore, further work was performed to enzymatically synthesise mRNA containing N1-methylpsuedouridine instead of uridine. The chromatograms are shown in Figure 1A and demonstrate that the AEX HPLC separates all 4 NTPs including the m1Ψ-UTP and the chemically modified mRNA. It is interesting to note that even on the rapid gradient used in this study, a change in retention time of the m1Ψ-UTP mRNA from the unmodified mRNA is observed (see Figure 2A), highlighting an alteration in the overall charge or hydrophilicity of the mRNA. This is consistent with the earlier elution of the m1Ψ-UTP compared to UTP (see Figure 1A) and demonstrates the influence of sequence on the separation of large RNA molecules using AEX.

In addition to testing how the inclusion of m1Ψ-UTP affects the mRNA peak in the chromatography, a variety of mRNAs with different lengths were assessed. Figure 2B shows the comparison between the peaks produced by mRNAs for eGFP (∼930 nt), COVID spike protein (C-Spike ∼4,300 nt) and NanoLuc (NLuc, ∼870 nt). The results show that all three mRNAs exhibit a peak within the 1.1–1.3 M NaCl window designed for elution of IVT products. Consistent with previous data shown for the different retention times of unmodified and modified (m1Ψ) mRNA, the order of elution of the mRNA is not related to the length of the mRNA and reflects the overall hydrophilicity of the RNA which is influenced by the sequence, potential secondary structure and charge density of the mRNA.

### 3.2 Quantification of mRNA and NTPs

Quantification of the mRNA and NTPs was performed in conjunction with external calibration curves. The linearity of the AEX HPLC assay was evaluated using a standard calibration curve with concentrations of NTPs ranging from 0.5–100 µM and amount of eGFP mRNA ranging from 1–530 ng. LOD and LOQ for eGFP mRNA were determined as 7.8 ng and 25.9 ng respectively. LOD for each of the individual NTPs ranged from 4.0–7.6 pmoles and LOQ ranged from 13.2–25.2 pmoles. These standards were prepared in triplicate and evaluated over the course of 3 days. In addition, the method robustness was tested by assessing the relative standard deviation (RSD) of the retention time for 100 µM NTPs and 530 ng eGFP mRNA. Intra- and inter-day RSDs were calculated for nine separate measurements over one or 3 days respectively. The results are summarised in Table 1.

**TABLE 1:** Retention time stability and linearity data.

Analyte	Retention time, RSD/%		Calibration curve, *R* ^2^
	Intra-day	Inter-day	
CTP	0.30	1.5	0.9997
ATP	0.24	1.3	0.9995
UTP	0.13	1.3	0.9996
GTP	0.09	1.2	0.9997
eGFP mRNA	0.02	0.11	0.9845
No. reps	9	3	3
No. days	1	3	3
Tot. reps	9	9	9

**FIGURE 3:**
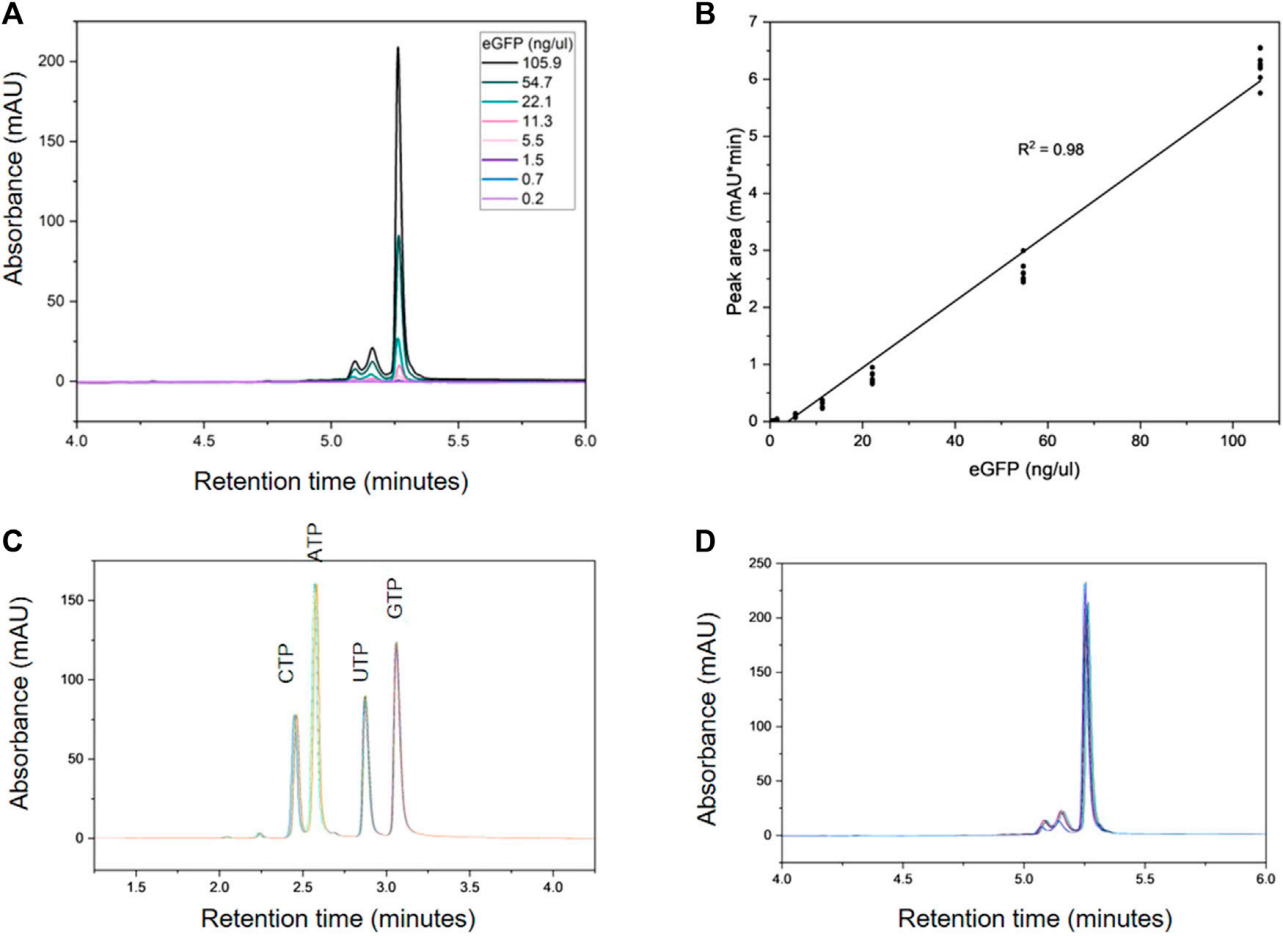
Quantification of mRNA and NTPs using AEX HPLC. **(A)** AEX chromatograms produced by a series dilution of eGFP mRNA (0.2–105.9 ng). **(B)** Calibration curve produced from the eGFP dilution series in triplicate over 3 days. Overlays of the intra-day analyses of 100 µM NTPs and 530 ng eGFP (n = 9) are shown in **(C)** and **(D)** respectively.

Figure 3A shows the HPLC analysis of the dilution series used to generate the calibration curve for eGFP mRNA, which is shown in Figure 3B. The slope demonstrates good linearity with a correlation coefficient (*R* ^2^) of 0.98. Each of the NTPs produced a calibration curve with an *R* ^2^ of 1.0; the dilution series and resulting calibration curves for the NTPs are shown in Supplementary Figure S3. Intra-day variability of retention times for NTPs and mRNA was ≤0.3%. Inter-day variability was higher for the retention time of the NTPs at ≤1.5% however the mRNA retention time variability remained at 0.11%.

### 3.3 Analysis of IVT reactions using AEX HPLC to determine mRNA yield and NTP consumption

Recent studies have demonstrated the application of HPLC monitoring of consumption of NTPs with concomitant production of mRNA, to optimise the yield of mRNA production (batch and fed batch processes) (Pregeljc et al., 2023). However, under the HPLC conditions employed no separation of CTP/UTP was observed, preventing accurate quantification of CTP/UTP consumption. Following optimisation of the AEX HPLC method for the analysis of IVT reactions, further work was performed to enable high throughput analysis and quantification of mRNA and NTPs directly from the IVT reactions. Quantification of the mRNA and NTPs was performed using external standards (C-Spike mRNA and separately NTPs) to generate separate calibration curves from both mRNA and NTPs–the dilution series and curve for C-Spike is shown in Supplementary Figure S4.

**FIGURE 4:**
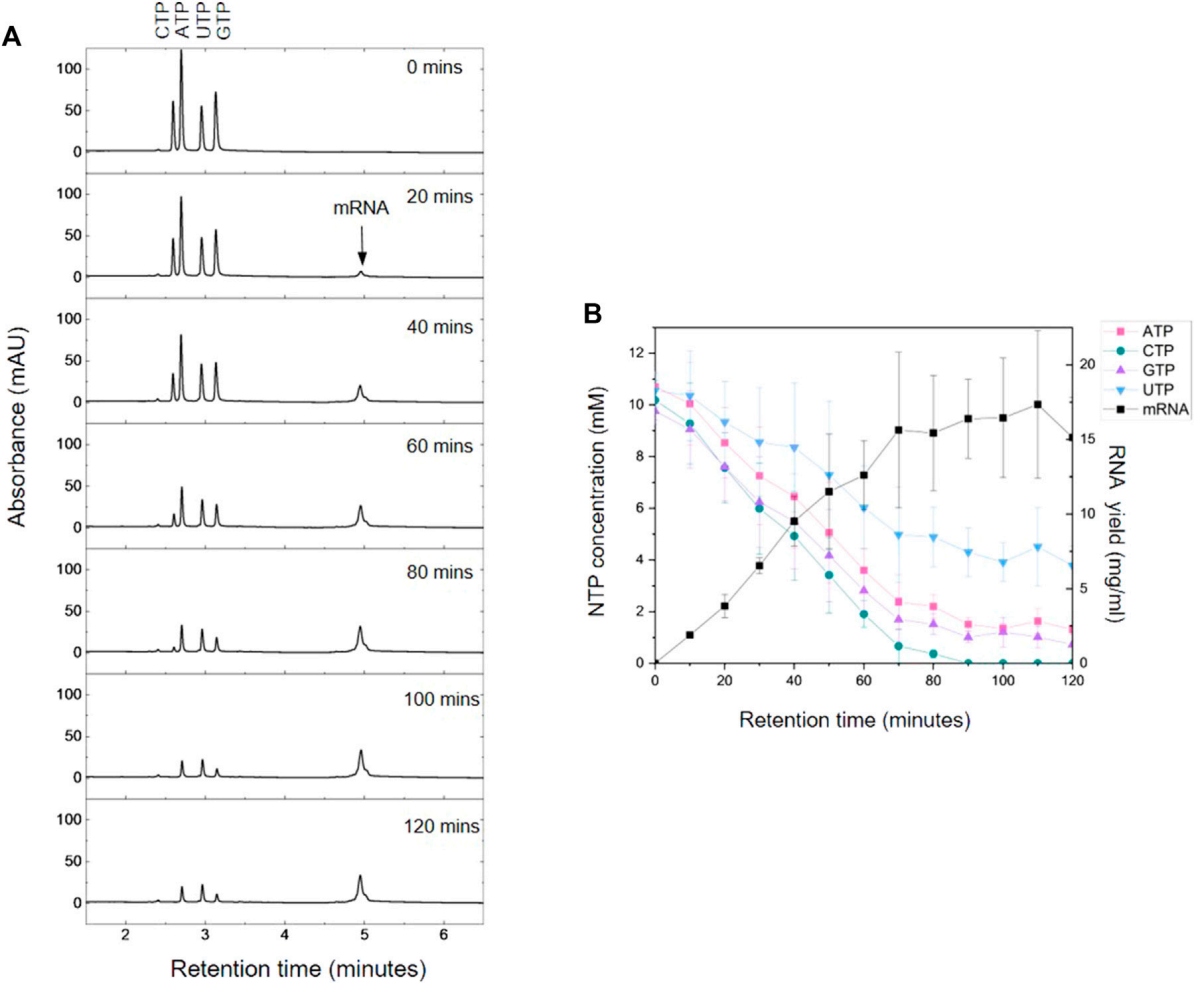
AEX HPLC analysis of the kinetics of mRNA production. **(A)** AEX chromatograms produced by time points taken every 20 min during an IVT reaction for C-Spike mRNA. **(B)** NTP concentration and mRNA yields determined using HPLC analysis in conjunction with standard curves for NTPs and mRNA. Mean and SD are plotted of n = 3 biological replicates.

The AEX HPLC enables direct analysis of the IVT reaction mixture with no sample preparation or purification required. To demonstrate the application of IVT analysis, samples were taken from selected time points (every 10 min, 0–2 h) from an IVT reaction producing C-Spike mRNA. The resulting chromatograms are shown in Figure 4A and corresponding quantification of the mRNA and NTPs is shown in Figure 4B. The results show the increase in synthesis of mRNA overtime and corresponding consumption of NTPs during the IVT reaction. The results show that the IVT reactions reached a maximum mRNA yield within approximately 70 min, furthermore, the results show that this plateau in mRNA production coincided with a significant drop of NTP concentration to approximately 10%–20% of the starting value for ATP/CTP/GTP. However, the concentration of UTP remained higher at approximately 50% of the starting value. The results also show that by 90 min all the CTP from the IVT reaction was essentially depleted. These results are consistent with the nucleotide composition of the C-Spike mRNA (31% C, 26% A, 19% U, 25% G) reflecting the demand for the corresponding NTPs during the synthesis of the mRNA. The ability to rapidly quantify all NTPs can be used to ensure mRNA synthesis is not inhibited by consumption or depletion of specific NTPs during the IVT reaction. This allows optimisation of the NTP ratios based on the target sequence to increase yield and reduce the frequency of miss-incorporation of NTPs. Efficient use of NTPs (especially m1Ψ-UTP) and capping analogues can substantially reduce manufacturing costs (Kis et al., 2022).

### 3.4 Monitoring mRNA purification using AEX HPLC

In addition to the quantification of mRNA synthesis and NTP consumption direct from IVT reactions, the developed AEX HPLC method was also used to monitor mRNA purification, in particular NTP/Clean Cap removal during the purification process. Prior to downstream applications and LNP formulation it is essential to remove IVT reaction components such as NTPs/Cap reagents, salts, proteins, *etc.*, which is required for accurate quantification using UV spectrophotometry of the purified mRNA drug substance. Therefore, the AEX HPLC method utilised in this study offers a rapid approach to monitor NTP/Cap analogue removal and mRNA yield during downstream purifications.

**FIGURE 5:**
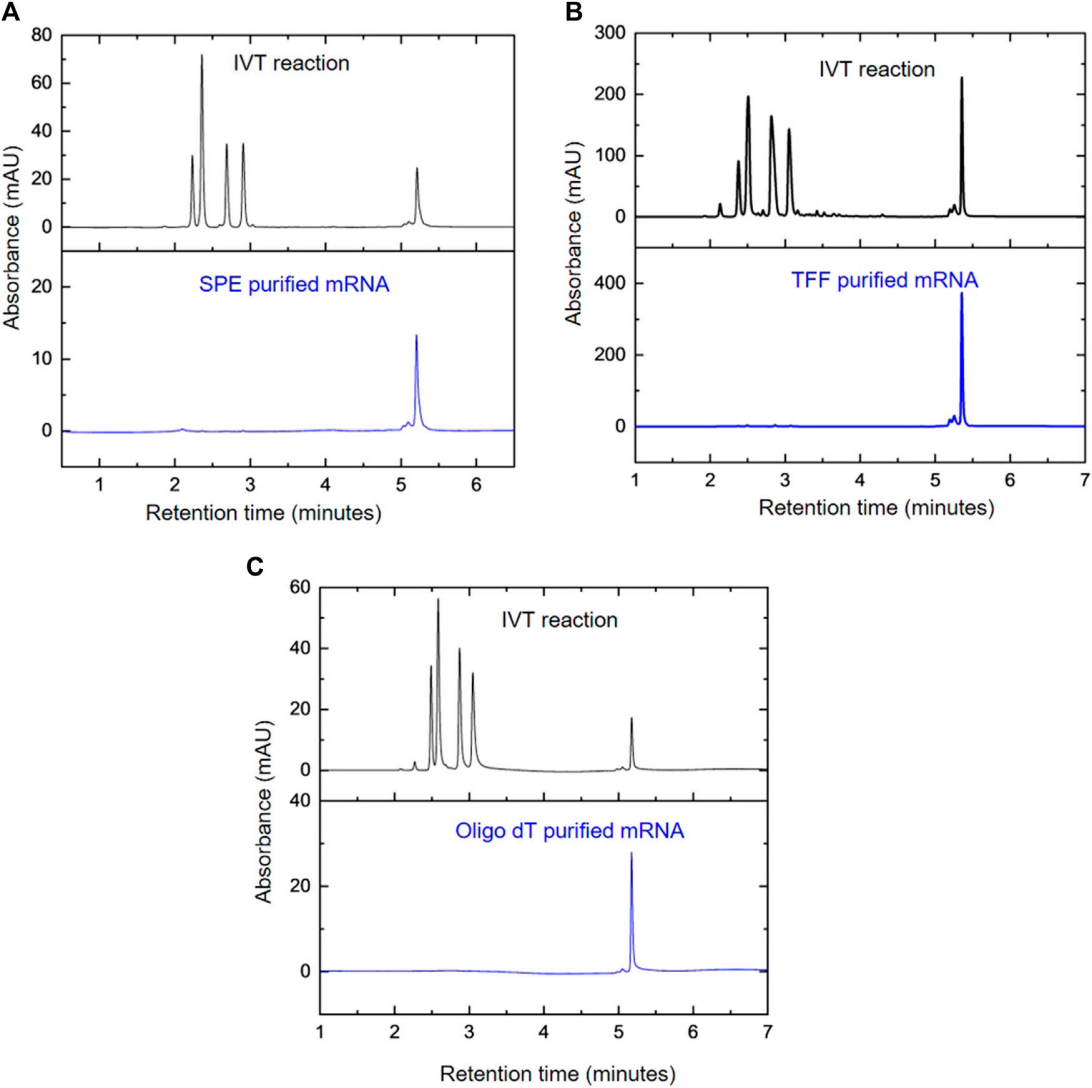
Monitoring mRNA purifications using AEX HPLC. AEX chromatograms comparing the IVT products before and after purification using; **(A)** SPE, **(B)** TFF diafiltration and **(C)** oligo-dT affinity chromatography.

To demonstrate the application of the AEX HPLC method, a variety of downstream purification methods were used to purify the mRNA prior to analysis. This included solid phase extraction (SPE) using silica membranes, TFF and oligo-dT affinity chromatography. The small scale purification using SPE is shown in Figure 5A and demonstrates the effective removal (>99%) of NTPs. Larger scale purification, *via* TFF in diafiltration mode and oligo-dT chromatography is demonstrated in Figures 5B, C respectively. The results also show the successful purification and removal of residual NTPs (>96% and >99.9% respectively).

This AEX HPLC method provides valuable information for developing kinetic models of the IVT unit operation and mass-balance models for downstream purification unit operations (van de Berg et al., 2021). These models can lay the foundations for establishing digital-twins and soft sensors, and for promoting accelerated production process development and automation of the final manufacturing process. Moreover, these models can be integrated into a Quality by Digital Design framework. Based on this, a design space can be defined for developing and manufacturing a wide range of RNA vaccines and therapeutics using the same platform process (Daniel et al., 2022).

## 4 Conclusion

mRNA has emerged as an important new class of therapeutic. Optimisation of the mRNA manufacturing process enables significant increases in mRNA yield and quality of the mRNA drug substance produced. Rapid analysis of IVT reactions and the mRNA generated during manufacturing, including downstream processing, using on-line or at-line analytical methods provides critical information for the optimisation of the mRNA manufacturing process. In this study we have developed a rapid method for the analysis of IVT reactions using AEX HPLC. This method enables quantification of each individual NTP, Cap analogue, mRNA and residual DNA, with separations achieved in <6 min. This method has been successfully used to measure mRNA yield, NTP consumption and mRNA purification in high throughput studies. This method was then utilised to monitor the progress of an IVT reaction for the production of mRNA. Here, it was possible to quantify the production of mRNA alongside the consumption of NTPs demonstrating that under the conditions used the IVT reaction, mRNA synthesis was complete at 70 min, in conjunction with significant consumption of NTPs (80%–90% for ATP/GTP/CTP) at this time. Furthermore, the AEX HPLC analysis was used to provide impurity analysis of the mRNA product following downstream purifications using SPE, TFF and oligo-dT affinity chromatography. The application of at-line analytics can provide the critical insight required for optimisation of the mRNA manufacturing process, enabling optimization of individual IVT reaction components, such as concentration of Mg2+, plasmid, NTP concentration, as well as adjusting the reaction parameters in near real-time. Therefore, this AEX HPLC method provides valuable information for developing kinetic models of the IVT unit operation and mass-balance models for downstream purification unit operations.

## Data Availability

The raw data supporting the conclusion of this article will be made available by the authors, without undue reservation.
