# Supplementary material for: Anion exchange HPLC monitoring of mRNA in vitro transcription reactions to support mRNA manufacturing process development
Source: Front Mol Biosci. 2024 Mar 7;11:1250833. doi: 10.3389/fmolb.2024.1250833 (PMC10955092; doi:10.3389/fmolb.2024.1250833)
Supplement: Supplementary file 1 [file Presentation1.PPTX]

## Slide 1
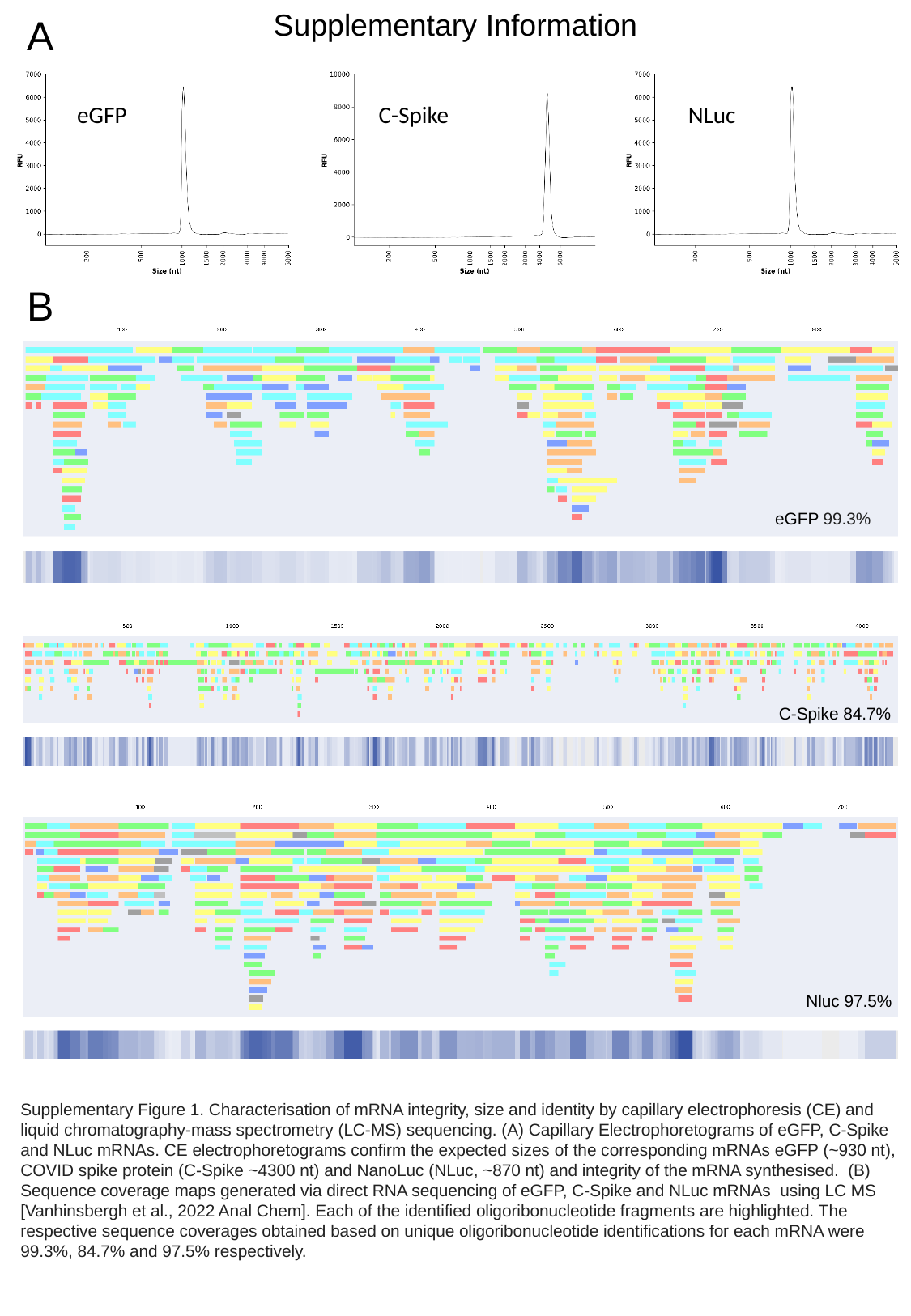

Supplementary Information
A
eGFP
C-Spike
NLuc
B
eGFP 99.3%
C-Spike 84.7%
Nluc 97.5%
Supplementary Figure 1. Characterisation of mRNA integrity, size and identity by capillary electrophoresis (CE) and liquid chromatography-mass spectrometry (LC-MS) sequencing. (A) Capillary Electrophoretograms of eGFP, C-Spike and NLuc mRNAs. CE electrophoretograms confirm the expected sizes of the corresponding mRNAs eGFP (~930 nt), COVID spike protein (C-Spike ~4300 nt) and NanoLuc (NLuc, ~870 nt) and integrity of the mRNA synthesised. (B) Sequence coverage maps generated via direct RNA sequencing of eGFP, C-Spike and NLuc mRNAs using LC MS [Vanhinsbergh et al., 2022 Anal Chem]. Each of the identified oligoribonucleotide fragments are highlighted. The respective sequence coverages obtained based on unique oligoribonucleotide identifications for each mRNA were 99.3%, 84.7% and 97.5% respectively.

## Slide 2
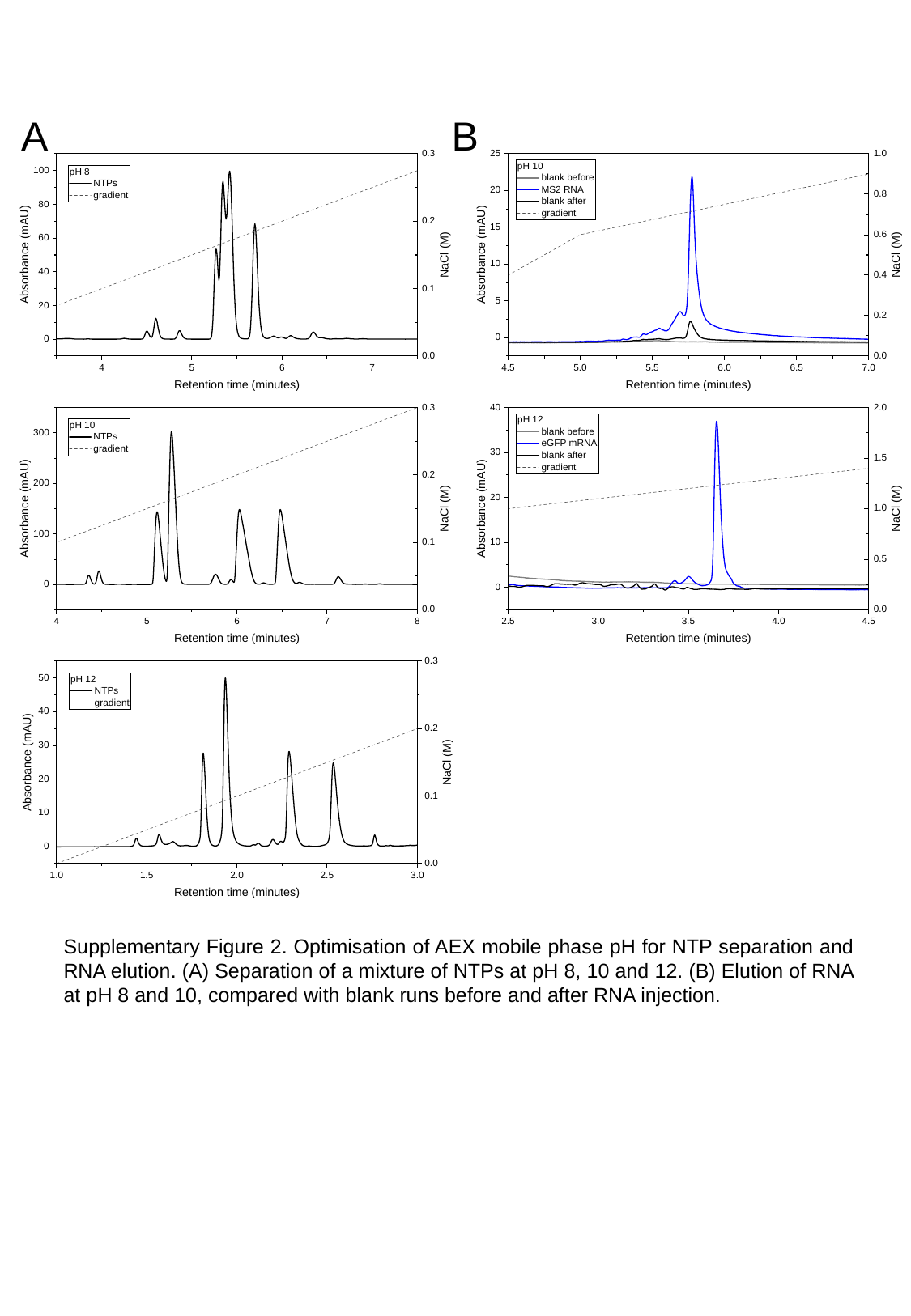

B
A
Supplementary Figure 2. Optimisation of AEX mobile phase pH for NTP separation and RNA elution. (A) Separation of a mixture of NTPs at pH 8, 10 and 12. (B) Elution of RNA at pH 8 and 10, compared with blank runs before and after RNA injection.

## Slide 3
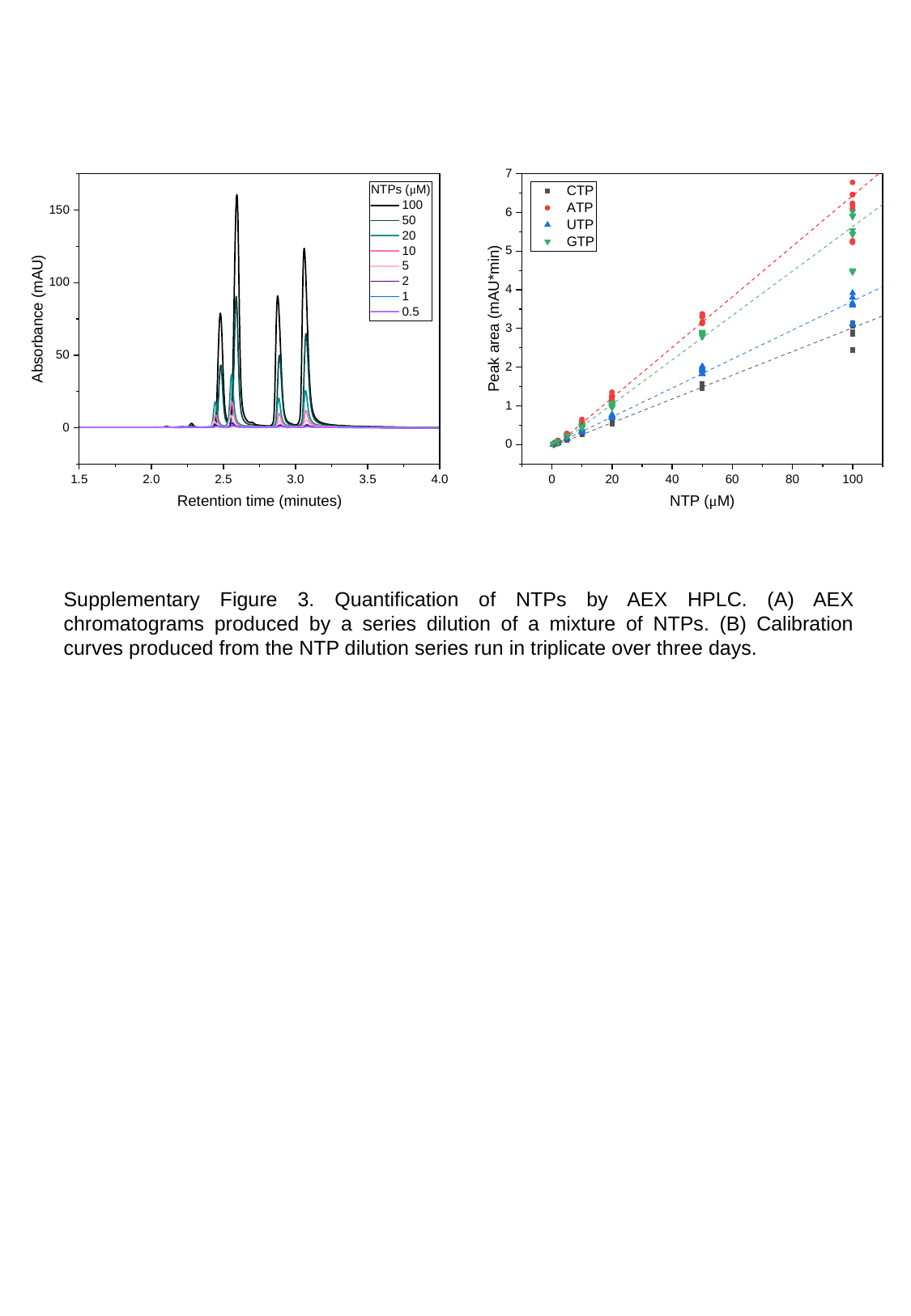

Supplementary Figure 3. Quantification of NTPs by AEX HPLC. (A) AEX chromatograms produced by a series dilution of a mixture of NTPs. (B) Calibration curves produced from the NTP dilution series run in triplicate over three days.

## Slide 4
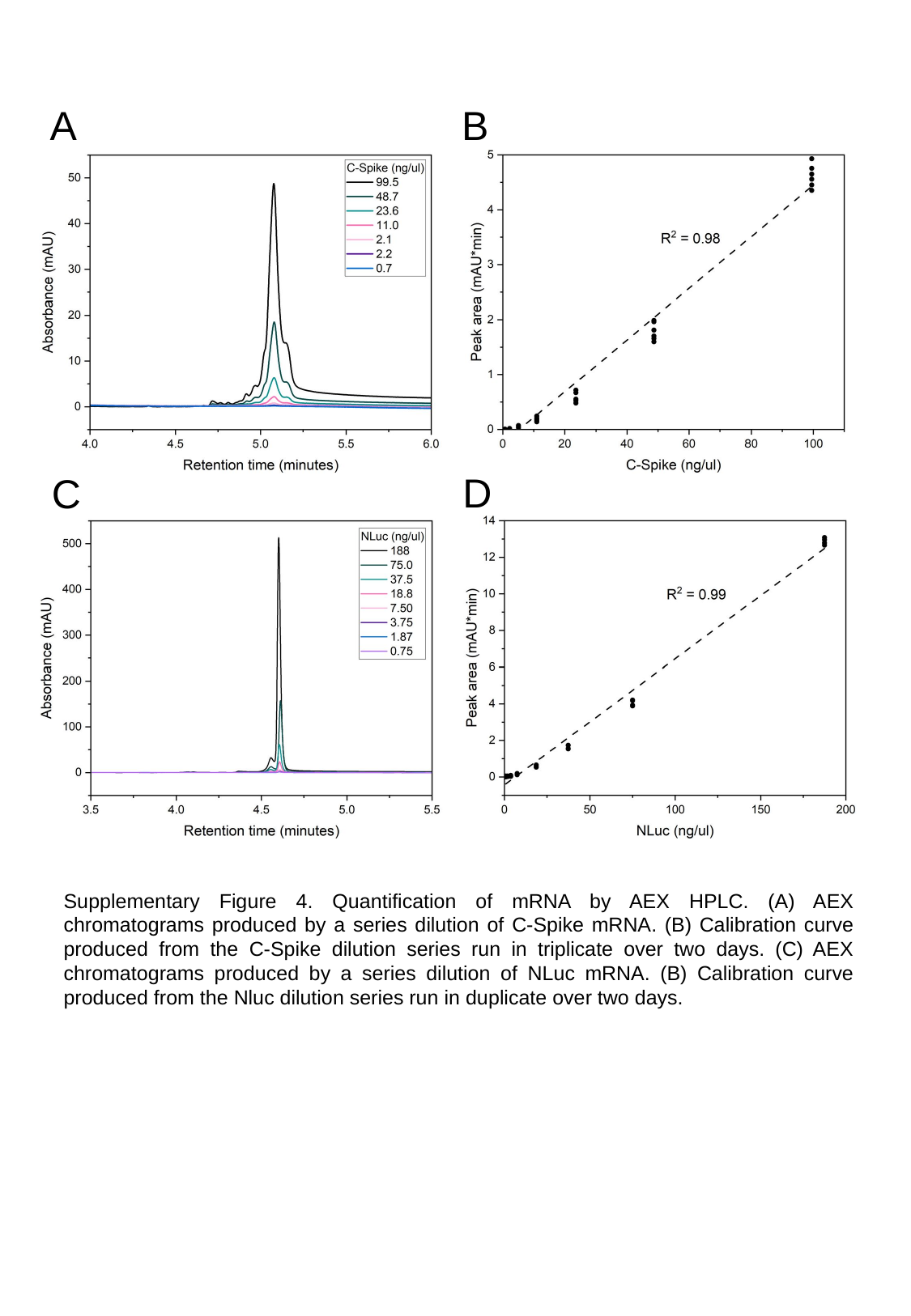

A
B
D
C
Supplementary Figure 4. Quantification of mRNA by AEX HPLC. (A) AEX chromatograms produced by a series dilution of C-Spike mRNA. (B) Calibration curve produced from the C-Spike dilution series run in triplicate over two days. (C) AEX chromatograms produced by a series dilution of NLuc mRNA. (B) Calibration curve produced from the Nluc dilution series run in duplicate over two days.
